# Sociodemographics and Attrition in Children With Osteosarcoma Enrolled in the AOST0331 Clinical Trial

**DOI:** 10.1001/jamanetworkopen.2026.3666

**Published:** 2026-03-27

**Authors:** Daniel J. Zheng, Natalie DelRocco, Ruxu Han, Mark Krailo, Rahela Aziz-Bose, Kristine A. Karvonen, Colleen A. Kelly, Haley Newman, Puja J. Umaretiya, Lenka Ilcisin, Damon R. Reed, Richard Gorlick, Katherine Janeway, Kira Bona

**Affiliations:** 1 Division of Oncology, Children’s Hospital of Philadelphia, Philadelphia, Pennsylvania; 2 Children’s Oncology Group, Monrovia, California; 3 Department of Population and Public Health Sciences, Keck School of Medicine, University of Southern California, Los Angeles; 4 Dana-Farber/Boston Children’s Cancer and Blood Disorders Center, Harvard Medical School, Boston, Massachusetts; 5 Public Health Sciences Division, Fred Hutchinson Cancer Center, Seattle, Washington; 6 Department of Pediatrics, UT Southwestern Medical Center, Dallas, Texas; 7 Division of Pediatric Surgery, University of Iowa, Iowa City; 8 Department of Pediatrics, Memorial Sloan Kettering Cancer Center, New York, New York; 9 Department of Pediatrics, The University of Texas MD Anderson Cancer Center, Houston

## Abstract

This cohort study examines whether participant attrition differed from enrollment to randomization by sociodemographics among children with localized osteosarcoma enrolled in a randomized clinical trial with delayed randomization.

## Introduction

Risk of selection bias can occur across the continuum of clinical trial conduct (Figure); however, existing literature has mostly evaluated disparities in initial enrollment. Many trials use a staged-consent process to reduce patient information overload, but it is unknown whether this delayed randomization design results in biased attrition (ie, participant dropout following initial consent).^1^ Ensuring ultimate generalizability of randomized trial end points is critical for research translation. We evaluated whether attrition differs by sociodemographics among patients with localized osteosarcoma enrolled at a US site in a phase 3, pediatric cooperative group trial with delayed randomization.

**Figure.**
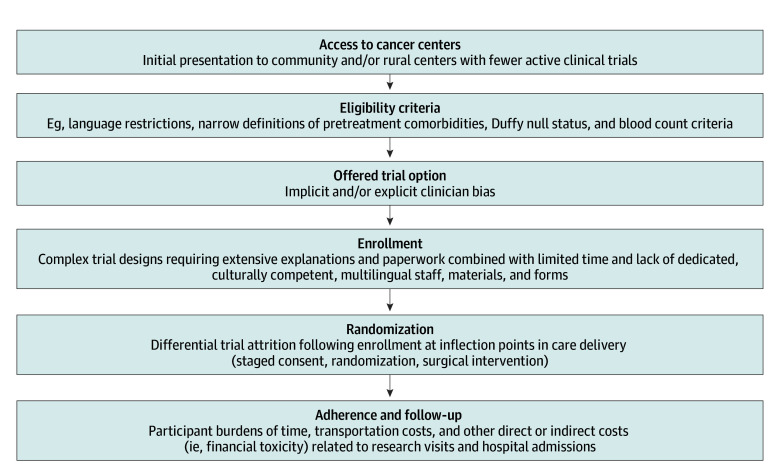
Inflection Points Along the Clinical Trial Continuum That May Lead to Nonrepresentative Participation^5,6^

## Methods

This cohort study was a secondary analysis of data from Children’s Oncology Group AOST0331, a randomized clinical trial that enrolled patients with newly diagnosed osteosarcoma from 2005 to 2011 and assessed 3 chemotherapy groups. Participants consented to enrollment prior to week 1 of induction chemotherapy and completed a second consent to randomization after week 11 surgery if they were confirmed to be free of detectable disease at that time.^2^ AOST0331 was approved by each site’s institutional review board. Participants provided written informed consent and assent for trial enrollment and future use of data. This analysis was restricted to eligible patients aged 21 years or younger with localized disease who enrolled and initiated induction chemotherapy at 146 US Children’s Oncology Group centers.^3^ This study is reported following the STROBE reporting guideline.

We compared sociodemographic characteristics between trial participants who consented to randomization vs those who were not randomized (including declined consent) in the absence of death or a disease event. Trial-collected sociodemographic data included age, sex, race and ethnicity (extracted from case report forms), household-level poverty exposure (proxied by insurance) and neighborhood-level poverty exposure (proxied by residential zip code).^3^ Additional methodologic and statistical details are available in eMethods in Supplement 1. A 2-sided *P* < .05 was considered statistically significant for all analyses. Data were analyzed from February to September 2025.

## Results

Among 758 candidate patients, 52 patients died or had a disease event prior to randomization. There were 278 patients (37%) without an event who were not randomized, and 428 patients (56%) were randomized, yielding an analytic cohort of 706 patients (median [IQR] age, 14 [11-16] years; 400 [57%] male), including 20% Hispanic, 16% Non-Hispanic Black, 55% non-Hispanic White, 5% non-Hispanic other patients (Table). There were no differences in consent to randomization by any examined characteristics, except age (Table). In univariable and multivariable analyses, older patients were 5% less likely to be randomized for every 1-year increase in age at enrollment (odds ratio; 0.95; 95% CI, 0.91-0.99; *P* = .03). Primary results did not differ in sensitivity analyses.

**Table.** Trial Attrition by Sociodemographic Characteristics

Characteristic	Patients, No. (%)		*P* value for equality of distribution^a^	OR for randomization (95% CI)^b^	*P* value for difference in randomization
	Not randomized (n = 278)	Randomized (n = 428)			
Sex					
Male	150 (54)	250 (58)	.25	1.22 (0.88-1.70)	.23
Female	128 (46)	178 (42)		1 [Reference]	NA
Age at enrollment, y^c^					
Median (IQR)	14 (4)	13 (5)		0.95 (0.91-0.99)	.03
Child	81 (29)	159 (37)	.02	NA	NA
Adolescent	150 (54)	221 (52)		NA	NA
Adult	47(17)	48 (11)		NA	NA
Race and ethnicity^d^					
Hispanic	61 (22)	83 (19)	.36	0.89 (0.58-1.36)	.58
Non-Hispanic Black	38 (14)	73 (17)		1.33 (0.83-2.12)	.23
Non-Hispanic White	150 (54)	240 (56)		1 [Reference]	NA
Non-Hispanic other	18 (6)	19 (4)		0.60 (0.29-1.24)	.17
Missing	11 (4)	13 (3)		NA	NA
Primary tumor site					
Axial	14 (5)	9 (2)	.09	1 [Reference]	NA
Proximal humerus or femur	36 (13)	50 (12)		1.68 (0.63-4.47)	.30
Other limb	228 (82)	369 (86)		2.09 (0.87-5.03)	.10
Neighborhood poverty					
No	190 (68)	313 (73)	.12	1 [Reference]	NA
Yes	87 (31)	110 (26)		0.73 (0.50-1.06)	.10
Missing	1 (<1)	5 (1)		NA	NA
Household poverty					
No	187 (67)	304 (71)	.47	1 [Reference]	NA
Yes	73 (26)	104 (24)		0.89 (0.61-1.31)	.55
Missing	18 (6)	20 (5)		NA	NA

Abbreviations: NA, not applicable; OR, odds ratio.

^a^ Univariate Pearson χ^2^ test.

^b^ Logistic regression model.

^c^ Age was treated as a linear continuous variable in the multivariable model, and sensitivity analysis showed similar results when treated as categorical variable (adolescent vs child, OR, 0.74; 95% CI, 0.51-1.06; *P* = .10; adult vs child, OR, 0.53; 95% CI, 0.32-0.89; *P* = .02).

^d^ Race and ethnicity were obtained from trial case report forms. Non-Hispanic other race and ethnicity includes American Indian or Alaska Native, Asian, Native Hawaiian or other Pacific Islander, and unknown (distinct from not reported).

## Discussion

In this cohort study using data from a large phase 3 trial for localized pediatric osteosarcoma that used staged consent to delayed randomization, we did not observe disparities in trial attrition prior to randomization based on sex, race and ethnicity, or poverty exposures. These results suggest that this study design, in which randomization occurred nearly 2 months after consent to enrollment, largely preserved the representativeness of the enrolled cohort. The observed increased attrition prior to randomization among older children warrants further investigation in the context of well-described outcome disparities for adolescent and young adult patients.^4^ This study’s limitations include restricting to results from a single trial and cancer type and heterogeneity in the other race and ethnicity group. While we focused on staged-consent trial design as a potential contributor to biased participation, prerandomization attrition is 1 of many time points where participation bias has the potential to impact trial generalizability, inclusion, and equity (Figure). Evaluation of multilevel strategies to optimize representative participation throughout the trial continuum—including modified eligibility criteria, decentralized trial delivery, patient navigators, multilingual staff and documents, and staff training in cultural competency and implicit bias—is key to maximize the benefits of clinical trial research.^5,6^

## Notes

Supplement 1. eMethods.

Supplement 2. Data Sharing Statement
