# Supplementary material for: Sociodemographics and Attrition in Children With Osteosarcoma Enrolled in the AOST0331 Clinical Trial
Source: JAMA Netw Open. 2026 Mar 27;9(3):e263666. doi: 10.1001/jamanetworkopen.2026.3666 (PMC13032149; doi:10.1001/jamanetworkopen.2026.3666)
Supplement: Supplement 1. — eMethods. [file jamanetwopen-e263666-s001.pdf]

Supplemental Online Content

Zheng DJ, DelRocco N, Han R, et al. Sociodemographics and attrition in children with osteosarcoma enrolled in the AOST0331 clinical trial. *JAMA Netw Open*. 2026;9(3):e263666. doi:10.1001/jamanetworkopen.2026.3666

eMethods.

This supplemental material has been provided by the authors to give readers additional information about their work.

© 2026 Zheng DJ et al. *JAMA Network Open*.

eMethods. Supplemental Methods

AOST0331 enrolled patients with newly diagnosed osteosarcoma from 2005–2011 in a randomized clinical trial of three chemotherapy arms. Participants consented to trial enrollment prior to week 1 of induction chemotherapy, and completed a second consent to randomization following week 11 surgery if they were confirmed to be free of detectable disease at that time.[1] AOST0331 was approved by each local site's institutional review board. Participants provided written informed consent and assent for trial enrollment and future use of data. This analysis was restricted to eligible patients ≤21 years with localized disease who enrolled and initiated induction chemotherapy at US Children's Oncology Group (COG) centers. Detailed eligibility and a flow diagram depicting the analytic cohort was previously published in manuscript text and a corresponding flow diagram (Figure 1, *Ilcisin et al 2024*).[2]

We compared sociodemographic characteristics between trial participants who consented to randomization following week 11 versus those who were not randomized (including declined consent) in the absence of death or a disease event. Disease events included progression of disease or secondary malignant neoplasm. Trial collected sociodemographic data included age in years, sex (male vs. female), race/ethnicity, household-poverty exposure proxied by insurance (public only vs. private/other) at trial enrollment, and neighborhood-poverty exposure proxied by US Census-defined high poverty (>20% of persons living below 100% federal poverty level) vs. low poverty (≤20%) residential zip code at trial enrollment. Race and ethnicity were obtained from trial case report forms. Case report forms are completed at the institutional level by clinical research assistants; the process by which the data entered are collected are not shared with COG. We report race and ethnicity as per US Office of Management and Budget guidelines. A combined race/ethnicity variable for this study was operationalized for analyses as Hispanic, non-Hispanic Black, non-Hispanic Other, non-Hispanic White. Non-Hispanic Other race/ethnicity included Native Hawaiian or other Pacific Islander, Asian, American Indian or Alaska Native, and Unknown (distinct from "Not Reported"). Private/other insurance included any commercial insurance including dual coverage with commercial and public options, as well as military insurance.

Categorical variables were reported as frequencies and percentages stratified by variable of interest. Continuous variables were described using median and interquartile range defined as the difference between the 25th and 75th percentiles. Associations between the binary primary outcome (patient randomization) and sociodemographic characteristics were explored in univariable and multivariable analyses. The patient demographics, disease characteristics, and poverty exposures (henceforth referred to as the "covariates") to be examined in univariable and multivariable models were defined *a priori* based on clinical expertise. For univariable analyses, Pearson Chi-squared tests were used to test the null hypothesis of independence between randomization and covariates.

To assess the potential association between odds of randomization and covariates simultaneously, the probability of randomization was modeled using a generalized linear model with logistic link (logistic regression model). The primary multivariable model treated age as a linear (continuous) based on clinical expertise. The assumption of linear functional form on age was assessed by fitting age as a restricted cubic spline with three basis knots equally spaced on the observed

© 2026 Zheng DJ et al. *JAMA Network Open*.

quantiles of the age distribution (allowing sufficient nonlinearity in the relationship between odds of randomization and age, if present) and testing via standard Wald test the null hypothesis that the coefficient associated with the nonlinear term in the model is no different from zero. No statistical evidence of nonlinearity in age was observed, and hence the linear functional form on age was deemed appropriate.

The assumption of additivity between the covariates included in the multivariable model was assessed by inclusion of four interaction terms defined *a priori* based on clinical expertise. Due to the large number of race categories and sparseness of size (impeding statistical model convergence), race was reduced to a two-category variable (White vs. Other) during this assumption check and estimated separate from ethnicity. The interaction terms were therefore fit between: (1) race and neighborhood poverty exposure, (2) race and household poverty exposure, (3) ethnicity and neighborhood poverty exposure, (4) ethnicity and household poverty exposure. A four degree of freedom likelihood ratio test was conducted for the null hypothesis that all coefficients associated with interaction terms were no different from zero. No statistical evidence of an interaction effect between race/ethnicity and poverty exposure was observed. Hence, the primary main effects only multivariable model deemed appropriate.

Three sensitivity analyses were additionally conducted:

1. An iteration of the multivariable model with age represented as a categorical variable. Age categories leveraged definitions from prior trial publications: child [male: 0-12 years; female: 0-11 years]; adolescent [male: 13-17 years; female: 12-16 years]; and adult [male: 18-20 years; female: 17-20 years].[1-3]
2. Using multiple imputation to account for missing data.
3. Including as a covariate of interest histological response of the primary tumor at the time of week 11 surgery.

A two-sided $P < .05$ was considered statistically significant for all analyses. No adjustment was made for multiple testing. Reporting followed the Strengthening the Reporting of Observational Studies in Epidemiology (STROBE) reporting guideline for cohort studies (see Supplemental STROBE checklist). All analyses were conducted using SAS Version 9.4 or R Statistical Software Version 4.5.1.

© 2026 Zheng DJ et al. *JAMA Network Open*.

References for Supplement:

1. Smeland S, Bielack SS, Whelan J, et al. Survival and prognosis with osteosarcoma: outcomes in more than 2000 patients in the EURAMOS-1 (European and American Osteosarcoma Study) cohort. *European journal of cancer*. 2019;109:36-50.
2. Ilcisin L, Han R, Krailo M, et al. Poverty, race, ethnicity, and survival in pediatric nonmetastatic osteosarcoma: a Children's Oncology Group report. *JNCI: Journal of the National Cancer Institute*. 2024;116(10):1664-1674.
3. Whelan J, Bielack S, Marina N, et al. EURAMOS-1, an international randomised study for osteosarcoma: results from pre-randomisation treatment. *Annals of oncology*. 2015;26(2):407-414.

© 2026 Zheng DJ et al. *JAMA Network Open*.
